# Sugar-sweetened beverages, relative grip strength, and psychological symptoms among rural adolescents in western China: a cross-sectional study

**DOI:** 10.3389/fnut.2025.1511256

**Published:** 2025-01-15

**Authors:** Yanni Zhang, Jianping Xiong, Rong Sun, Guangxin Chai, Li Xiong

**Affiliations:** ^1^Department of Physical Education, Yangtze University College of Arts and Sciences, Jingzhou, China; ^2^School of Physical Education, Jiangxi University of Finance and Economics, Nanchang, China; ^3^School of Physical Education and Health, Jiangxi Science and Technology Normal University, Nanchang, China; ^4^School of Physical Education and Health, Nanchang Institute of Science & Technology, Nanchang, China

**Keywords:** adolescents, grip strength, psychological symptoms, sugar-sweetened beverages, western China

## Abstract

**Background:**

The increasing prevalence of psychological symptoms in adolescents has become an important problem faced by all countries in the world. The increased sugar-sweetened beverages (SSB) consumption and the decreased muscle strength had a serious negative impact on adolescent health. However, previous studies have mainly focused on adolescents in developed countries and fewer studies have been conducted in developing countries, especially in rural areas of western China. This study aims to explore the association of sugar-sweetened beverages consumption, and relative grip strength with psychological symptoms among rural adolescents in western China.

**Methods:**

In this study, 11,018 adolescents aged 13–18 years from rural areas of Xinjiang and Tibet in western China were recruited using stratified randomized whole-cluster sampling in 2023. The participants were assessed for sugar-sweetened beverages consumption, relative grip strength, and psychological symptoms. Non-parametric tests, *t*-tests, logistic regression analyses, and ordered logistic regression analyses of generalized linear models were used to analyze the associations of sugar-sweetened beverage consumption and relative grip strength with psychological symptoms in adolescents.

**Results:**

The proportions of adolescents with sugar-sweetened beverages consumption of <1 times/week, 2–4 times/week, and >4 times/week in rural areas of western China were 34.6, 52.7, and 12.7%, respectively. The prevalence of adolescents’ emotional problems, behavioral problems, social adjustment difficulties, and psychological symptoms were 28.7, 27.0, 20.2, and 22.1%, respectively. The mean and standard deviation of grip strength among adolescents was assessed as (32.52 ± 10.13) kg and the relative grip strength was (0.60 ± 0.16) in rural areas of western China. Taking participants with sugar-sweetened beverages consumption <1 times/week group and relative grip strength at the fourth quartile as the reference, participants with SSB consumption >4 times/week and relative grip strength at the first quartile had the highest risk (OR = 2.77, 95% CI: 2.09–3.67, *p* < 0.001) of psychological symptoms.

**Conclusion:**

Elevated sugar-sweetened beverages consumption and decreased relative grip strength were associated with an increased prevalence of psychological symptoms. Prospective cohort studies are needed in the future to explore the causal relationships among SSB consumption, muscle strength, and psychological symptoms.

## 1 Introduction

In recent years, with the continuous development of science and technology, people’s lifestyles have changed dramatically, such as an increase in light physical activity behaviors, the prolongation of video screen time and static behaviors, and the continuous increase in sugar-sweetened beverages (SSB) consumption (1–3). These lifestyle changes have led to a sustained increase in obesity rates among adolescents, as well as a sustained decrease in muscle strength, which has led to a sustained increase in the prevalence of psychological symptoms, posing a serious threat to adolescent health development (4). Globally, a meta-analysis of 29 studies showed a significant increase in the prevalence of depression and anxiety symptoms among adolescents, posing a threat to adolescent health (5). Another analysis, which included 41 studies in 27 countries around the world, showed that the global prevalence of mental disorders was as high as 13.4%, with a negative impact on academic performance and quality of life in future adulthood (6). It was also found that an estimated 8.5% of adolescents globally suffered from depression and 11.6% suffered from anxiety before COVID-19, compared to 23.8 and 19.0%, respectively, after the epidemic, resulting in a serious disease burden for governments (7, 8). The same trend exists in developed countries. The study found that from 2011 to 2020, U.S. adolescent mental health-related office visits increased from 4.8 million to 7.5 million, with an average annual growth rate of 8.0%, which suggests that all types of disorders due to mental health problems among U.S. adolescents have increased rapidly over the past 10 years, emphasizing the need to urgently improve mental health research and interventions for adolescents (9). China, also a developing country, is no exception. The study shows that 21.4% of adolescents in China have psychosomatic symptoms, which hurt adolescent health (10). In addition, the prevalence of psychological symptoms among adolescents in rural areas of China is significantly higher than that in urban areas, and the prevalence of psychological symptoms among adolescents in western China, where the level of economic development is relatively backward, is also higher than that in eastern China, where the level of economic development is comparatively higher, which suggests that adequate attention should be paid to psychological health problems among adolescents in rural areas of western China. This shows that the mental health problems of adolescents in rural areas of western China should be given sufficient attention and concern (11, 12). It has also been shown that the occurrence of psychological symptoms in adolescence will have an impact on adult health, with significant trajectory effects (13). The occurrence of adolescent psychological symptoms is affected by a combination of factors, which are mainly related to dietary behavior and physical activity, of which SSB consumption has become one of the most important risk factors affecting the physical and mental health of adolescents, which is worthy of attention and concern (14).

According to surveys, SSB consumption among adolescents is increasing globally, posing a serious threat to adolescent health (15). An analysis of SSB consumption in 185 countries showed that 56 countries (30.3%) had adolescents consuming an average of ≥7 servings per week, which represents 238 million adolescents, or about 10.4% of the global population in this age group involved in higher frequency SSB consumption (16). A survey of U.S. adolescents also showed that 32.0% of U.S. adolescents aged 12–18 years had high SSB consumption and 47.9% had low SSB consumption and measures should be taken to improve the dietary behaviors of U.S. adolescents (17). A survey of Chinese adolescents also showed that adolescent SSB consumption was consistently elevated, with the main frequency of adolescent SSB consumption ranging from 1 to 3 times/week, and the average intake of SSB among adolescents aged 6–17 years was 193.8 g/d, which poses a serious threat to health (18). Of concern are the various types of adverse health effects associated with continued elevated SSB consumption (19). Increased SSB consumption is an important risk factor for obesity, chronic cardiovascular disease, type 2 diabetes mellitus, dental caries, and various types of cancers, resulting in a large disease burden for all countries (15, 20, 21). The reasons for the impact of SSB consumption on health are manifold. Firstly, excessive SSB consumption will lead to the development of obesity, which is an important risk factor for the development of various chronic diseases. Secondly, excessive SSB consumption will also lead to an inflammatory response in the body, which will lead to a decrease in immunity, a decrease in muscle strength, and psychological problems, thus posing many health threats (15, 20, 21). In addition, it has been found that increased SSB consumption will lead to the development of various types of psychological disorders, mainly in the form of a sustained increase in the prevalence of psychological symptoms, which will hurt mental health (22). It is noteworthy that SSB consumption is increasing in developing countries, especially in rural areas where economic development is less advanced, with negative impacts on mental health, which should be a cause for concern and attention (23). Increased SSB consumption also negatively affects muscle strength, posing a threat to adolescent health (24).

Grip strength, as an important indicator of upper limb muscle strength in adolescents, is associated with several health indicators and has been of great interest to researchers in various countries (25, 26). Studies have shown significant associations between adolescent grip strength levels and mental health, various chronic diseases, cancer, and the ability to influence adult health (27, 28). It is worth noting that absolute grip strength tends to ignore the effect of body weight on muscle strength. Therefore, to more accurately assess the influence of muscle strength on various health factors, in recent years, scholars have more widely used relative grip strength to assess the muscle strength of adolescents, to better eliminate the influence of body weight on muscle strength (29). The study shows that there is an association between RELATIVE grip strength and mental health in adolescents, and calls for the effective improvement of adolescents’ grip strength level to promote mental health development (30). It has also been found that relative grip strength can more accurately assess people’s depression and anxiety than absolute grip strength levels to more accurately assess people’s mental health (31–33).

Given the continued elevation of SSB consumption and the decline in relativistic grip strength levels, coupled with the associations that exist between SSB consumption and grip strength levels with adolescent mental health. It is necessary to analyze the correlations that exist between SSB consumption and relative grip strength and psychological symptoms in adolescents. To provide a reference for the prevention and intervention of adolescents’ psychological symptoms to better promote adolescents’ mental health. However, it is noteworthy that past studies have mainly focused on adult or elderly populations in developed countries, while relatively few studies have been conducted on adolescent populations in developing countries (34, 35). At the same time, no research has been conducted on adolescents in rural areas of western China, where economic development is relatively backward. Given the relatively backward economic development of rural areas in western China, coupled with the high prevalence of psychological symptoms among adolescents in rural areas, this study therefore focuses on adolescents in rural areas of western China. The purpose of this study was to analyze the associations between SSB consumption, and relative grip strength with psychological symptoms in adolescents, and to provide reference and theoretical support for adolescent mental health education and intervention in rural areas of western China.

## 2 Methods

### 2.1 Participants

This study used stratified random whole cluster sampling for participant sampling and assessment from September to November 2023 in the rural areas of Xinjiang and Tibet in the western region of China. Participant extraction was divided into the following steps: First, the rural areas of Xinjiang and Tibet in the western region of China were selected as the participant sampling areas for this study. Second, two rural areas were selected in each province, Hotan and Yining in Xinjiang and Nagchu and Linzhi in Tibet. Third, 4 secondary schools in each of the selected rural areas were chosen as the schools to be assessed. Fourth, according to grade level, 3 teaching classes were randomly selected for each grade level, and a total of 12 teaching classes were selected for each school as the classes to be assessed in this study. Adolescents in the classes who met the inclusion criteria served as participants in this study. The inclusion criteria for this study were: adolescents between the ages of 13 and 18 who were enrolled in school, whose fathers and mothers and the participants themselves were of rural household registration, and who gave informed consent to the participants and their parents and volunteered to be surveyed for this study. In the end, a total of 11,872 adolescents aged 13–18 years old in rural areas from 288 teaching classes were assessed in this study. After the assessment, 854 questionnaires that were damaged, lost, had a response rate of less than 70% and were missing major demographic information were excluded. Finally, 11,018 valid questionnaires were returned in this study, with a valid return rate of 92.81%.

**Figure 1:**
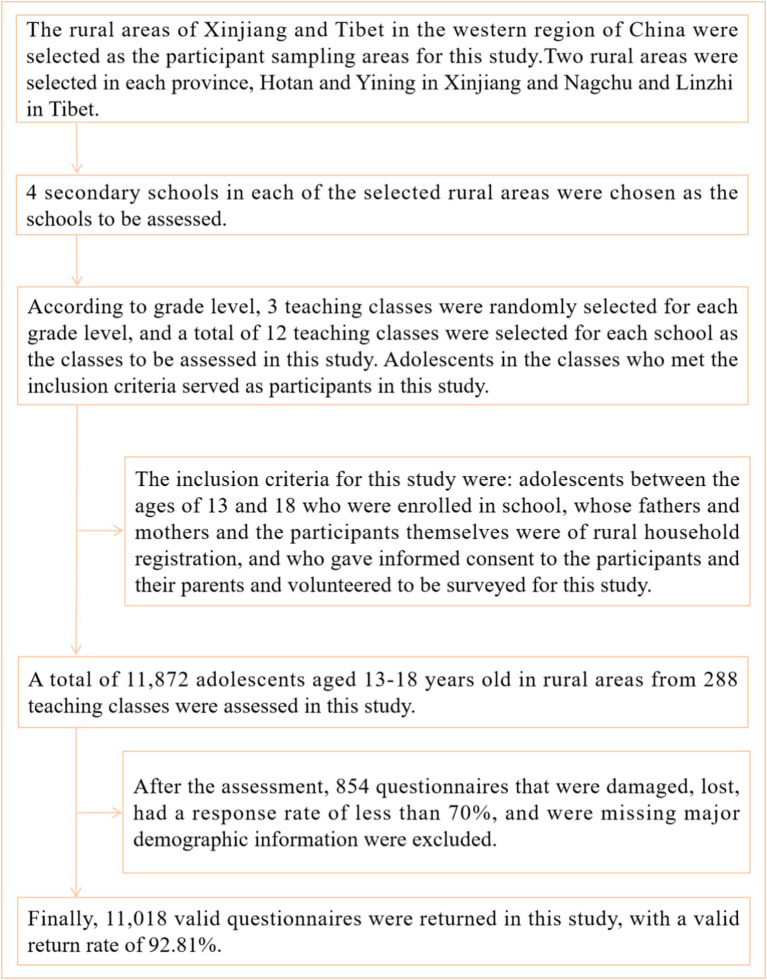
Sampling process of adolescent participants in rural areas of western China.

Figure 1 shows the sampling process of adolescent participants in this study in rural areas of western China. This study was conducted by the Declaration of Helsinki. Informed consent was obtained from parents or guardians before the assessment of participants in this study, and participants volunteered to be assessed for this study. Approved by the Human Ethics Committee of Jiangxi Science and Technology Normal University (IRB-JXSTNU-2022003).

### 2.2 SSB consumption assessment

In this study, SSB consumption among adolescents in rural areas of western China was assessed using the beverage intake questionnaire (BEVQ-15) (36). The EVQ-15 questionnaire is widely used in China for the assessment of SSB consumption and has good reliability and validity (37). The EVQ-15 questionnaire consists of 15 entries. It mainly assesses the frequency of SSB consumption, the amount consumed, and the types of SSB consumed by the participants in the past 1 month, and is calculated by using a fixed formula to derive (38). Based on the EVQ-15 questionnaire and in conjunction with the actual assessment of this study, the types of SSBs in this study included functional beverages, carbonated beverages, unsweetened colas, nut-based beverages, coffee, beer, milk with added sugar, and milk tea with added sugar. The amount assessed in this study was assessed according to the commonly used consumption amount, which was calculated using 8 ounces as 1 cup. The frequency of participants’ SSB consumption in this study was categorized into 3 types, respectively “≤1 times/week,” “2–4 times/week,” and “≥4 times/week.”

### 2.3 Relative grip strength assessment

The relative grip strength was calculated as absolute grip strength (kg)/body weight (kg). The assessment of absolute grip strength and body weight in this study was based on the testing instruments and methods required by the China National Survey on Students’ Constitution and Health (CNSSCH) (39). The absolute grip strength of the participants in this study was assessed using an electronic grip strength meter (Wanqing brand WCS-10000, China). Participants were asked to perform the test twice with the left hand and twice with the right hand. The average of the best performance of the left hand and the best performance of the right hand was used as the final absolute grip strength of the participants in this study. The results were evaluated to the nearest 0.1 kg (39). Participants were asked to assess absolute grip strength with their arms naturally hanging down in a standing position (39). The grip strength assessment can be done by adjusting the grip strength meter’s handle according to the size of one’s hand to guarantee the accuracy of the grip strength assessment results. Participants are required to do sufficient preparatory activities before the assessment to conduct the grip strength assessment more accurately (39).

Participants’ weight was assessed using an electronic weight scale (Omron HN – 289 model, China). The results were accurate to 0.1 kg, and participants were asked to empty their bowels and urine before the weight assessment. Participants were asked to wear light clothing for the weight assessment to ensure the accuracy of the results (39).

Grip strength and weight are assessed by trained and qualified staff. The assessment results are recorded on a questionnaire and alteration of the assessment results is strictly prohibited. Staff are required to calibrate the instruments before each day’s assessment to guarantee the accuracy of the results.

### 2.4 Psychological symptoms assessment

In this study, the prevalence of psychological symptoms among adolescents in rural areas of western China was assessed using the Brief Instrument on Psychological Health of Youths (BIOPHY) (40). The BIOPHY questionnaire consists of 15 items assessing various types of manifestations of psychological symptoms in the past 6 months. The BIOPHY questionnaire consists of three dimensions: emotional problems, behavioral problems, and social adjustment difficulties. The presence or absence of Psychological symptoms was based on the total score of the participant’s assessment. Each entry was categorized into six options, ranging from “more than 3 months” to “none or less than a week.” Participants were asked to choose one of these options based on their performance. Participants with symptoms lasting more than 1 month or more were defined as having a positive result, which was recorded as a score of 1, and vice versa. The scores for each dimension were summed to obtain a total psychological symptoms score. A score of <P90 was defined as the absence of psychological symptoms, and a score of ≥P90 was defined as the presence of psychological symptoms. This questionnaire has been widely used among Chinese adolescents (41, 42). The questionnaire has good reliability and validity with a Cronbach alpha coefficient of 0.93.

### 2.5 Covariates

The covariates in this study include the father’s education level, the mother’s education level, the family’s economic level, commuting to and from school, sufficient sleep, exercise duration, and BMI. The father’s education level and the mother’s education level are divided into junior high school and below, high school, college, and above. Family economic level is categorized into three types, which are <5,000 RMB/month, 5,000–10,000 RMB/month, and >10,000 RMB/month. Commuting to and from school was categorized into two types, positive approach, and negative approach. Sufficient sleep was categorized into two types based on relevant studies: No (<8 h/day), and Yes (≥8 h/day) (43). Exercise duration was categorized into three types based on the classification of related studies into <30 min/day, 30–60 min/day, and >60 min/day (44). BMI was calculated based on the height and weight of the participants. BMI was calculated using the formula weight (kg)/height (m)^2^.

### 2.6 Statistical analysis

The results of the continuous type of assessment in this study were expressed as mean and standard deviation (M ± SD). The results of the subtyped variables were expressed as percentages (%). A comparison of means between sexes and the presence of psychological symptoms was performed using a *t*-test. A comparison of basic characteristics and prevalence of psychological symptoms by subtype was performed using non-parametric tests. The analysis of the association between SSB consumption and relative grip strength and psychological symptoms was performed using logistic regression analysis and ordered logistic regression analysis. In logistic regression analysis, the presence of psychological symptoms was analyzed as the dependent variable, and the SSB consumption and relative grip strength quartiles were analyzed as the independent variables. Model 1 is crude, Model 2 adjusts age, father’s education level, mother’s education level, and family economic level based on Model 1, and Model 3 adjust commuting to and from school, sufficient sleep, exercise duration, and BMI based on Model 2. In ordered logistic regression analyses, the presence of psychological symptoms was analyzed as the dependent variable, and SSB consumption and relative grip strength quartiles combined across groups were analyzed as independent variables. Ordered logistic regression Model adjusts age, father’s education level, mother’s education level, family’s economic level, commuting to and from school, sufficient sleep, exercise duration, and BMI. Logistic regression analysis results are reported as ORs and 95% CIs, respectively. Data analysis was performed using SPSS 25.0 software. A two-sided test level of *p* < 0.05 was used.

## 3 Results

Table 1 shows the basic characteristics of adolescents in rural areas of western China. In this study, 11,018 (5,294 boys, 48.0%) adolescents aged 13–18 years in rural areas were assessed for SSB consumption, relative grip strength, and psychological symptoms. The mean age of the participants was (15.81 ± 1.60) years.

**Table 1:** Basic characteristics of adolescents aged 13–18 in rural areas of western China.

Variables	Boys	Girls	Total	*χ*2/*t*-value	*p*-value
*N*	5,294(48.0)	5,724(52.0)	11,018	8.064	0.041
Age (years)	15.77 ± 1.59	15.85 ± 1.60	15.81 ± 1.60	−2.661	0.008
Father’s education level [*N*(%)]				12.639	0.002
Junior high school and below	4,011(75.8)	4,349(76.0)	8,360(75.9)		
High School	1,030(19.5)	1,175(20.5)	2,205(20.0)		
College and above	253(4.8)	200(3.5)	453(4.1)		
Mother’s education level [*N*(%)]					
Junior high school and below	4,339(82.0)	4,748(82.9)	9,087(82.5)	6.402	0.041
High School	750(14.2)	804(14.0)	1,554(14.1)		
College and above	205(3.9)	172(3.0)	377(3.4)		
Family economic level [*N*(%)]				117.876	<0.001
<5,000 RMB/month	3,646(68.9)	4,425(77.3)	8,071(73.3)		
5,000–10,000 RMB/month	1,146(21.6)	995(17.4)	2,141(19.4)		
>10,000 RMB/month	502(9.5)	304(5.3)	806(7.3)		
Commuting to and from school [*N*(%)]					
Positive approach	2,355(44.5)	2,618(45.7)	4,973(45.1)	1.743	0.187
Negative approach	2,939(55.5)	3,106(54.3)	6,045(54.9)		
Sufficient sleep [*N*(%)]					
No (<8 h/day)	4,567(86.3)	5,113(89.3)	9,680(87.9)	24.109	<0.001
Yes (≥8 h/day)	727(13.7)	611(10.7)	1,338(12.1)		
Exercise duration [*N*(%)]					
<30 min/day	2,357(44.5)	3,364(58.8)	5,721(51.9)	299.637	<0.001
30–60 min/day	2,183(41.2)	1988(34.7)	4,171(37.9)		
>60 min/day	754(14.2)	372(6.5)	1,126(10.2)		
SSB consumption [*N*(%)]				179.982	<0.001
<1 times/week	1,530(28.9)	2,283(39.9)	3,813(34.6)		
2–4 times/week	2,939(55.5)	2,872(50.2)	5,811(52.7)		
>4 times/week	825(15.6)	569(9.9)	1,394(12.7)		
Relative grip strength quartiles [*N*(%)]				2543.605	
Q1	572(10.8)	2,176(38.0)	2,748(24.9)		
Q2	881(16.6)	1867(32.6)	2,748(24.9)		
Q3	1,576(29.8)	1,238(21.6)	2,814(25.5)		
Q4	2,265(42.8)	443(7.7)	2,708(24.6)		
Emotional problems [*N*(%)]	1,517(28.7)	1,649(28.8)	3,166(28.7)	0.032	0.859
Behavioral problems [*N*(%)]	1,466(27.7)	1,514(26.5)	2,980(27.0)	2.149	0.143
Social adjustment difficulties [*N*(%)]	1,120(21.2)	1,110(19.4)	2,230(20.2)	5.301	0.021
Psychological symptoms [*N*(%)]	1,197(22.6)	1,237(21.6)	2,434(22.1)	1.597	0.206
Height (M ± SD)	169.71 ± 8.37	159.90 ± 6.27	164.61 ± 8.84	69.962	<0.001
Weight (M ± SD)	58.27 ± 11.68	50.86 ± 8.33	54.42 ± 10.74	38.562	<0.001
BMI (M ± SD)	20.12 ± 3.22	19.86 ± 2.87	19.99 ± 3.04	4.483	<0.001
Grip strength (M ± SD)	38.79 ± 9.68	26.72 ± 6.41	32.52 ± 10.13	77.703	<0.001
Relative grip strength (M ± SD)	0.68 ± 0.16	0.53 ± 0.13	0.60 ± 0.16	51.691	<0.001

N, numbers; M, mean; SD, standard deviation; SSB, sugar-sweetened beverage; BMI, body mass index.

The results of this study showed that the proportions of adolescents with SSB consumption of <1 times/week, 2–4 times/week, and >4 times/week in rural areas of western China were 34.6, 52.7, and 12.7%, respectively. The difference in the detection rate of SSB consumption between sexes was statistically significant (*χ*^2^-value: 179.982, *p* < 0.001). The prevalence rates of emotional problems, behavioral problems, social adjustment difficulties, and psychological symptoms among adolescents in rural areas of western China were 28.7, 27.0, 20.2, and 22.1%, respectively. Adolescent grip strength was assessed as (32.52 ± 10.13) kg. Adolescent relative grip strength was (0.60 ± 0.16).

Table 2 shows a comparison of the prevalence of psychological symptoms among different categories of adolescents in rural areas of western China. The results showed that the prevalence of psychological symptoms among adolescents with SSB consumption <1 times/week, 2–4 times/week, and >4 times/week in rural areas of western China were 20.0, 21.1, and 31.9%, respectively, with statistically significant differences (*χ*^2^-value of 90.086, *p* < 0.001). The prevalence of psychological symptoms in adolescents with Relative grip strength quartiles for Q1, Q2, Q3, and Q4 was 26.1, 21.2, 21.0, and 20.1%, respectively, and the difference was statistically significant in comparison (*χ*^2^-value of 34.674, *p* < 0.001).

**Table 2:** Comparison of the prevalence of different categories of adolescent psychological symptoms in rural areas of western China.

Variables	Psychological symptoms		*χ*^2^/*t*-value	*P*-value
	No	Yes		
*N*	8,584	2,434		
Age (years)	15.84 ± 1.61	15.71 ± 1.53	3.708	<0.001
Height (M ± SD)	164.49 ± 8.86	165.04 ± 8.75	−2.672	0.008
Weight (M ± SD)	54.01 ± 10.45	55.86 ± 11.59	−7.525	<0.001
BMI (M ± SD)	19.86 ± 2.91	20.42 ± 3.45	−7.981	<0.001
Grip strength (M ± SD)	32.61 ± 10.02	32.19 ± 10.52	1.826	0.068
Relative grip strength (M ± SD)	0.61 ± 0.16	0.58 ± 0.17	6.721	<0.001
Sex [*N*(%)]			1.597	0.206
Boys	4,097(77.4)	1,197(22.6)		
Girls	4,487(78.4)	1,237(21.6)		
Father’s education level [*N*(%)]			0.412	0.814
Junior high school and below	6,502(77.8)	1858(22.2)		
High School	1729(78.4)	476(21.6)		
College and above	353(77.9)	100(22.1)		
Mother’s education level [*N*(%)]			4.280	0.118
Junior high school and below	7,069(77.8)	2018(22.2)		
High School	1,205(77.5)	349(22.5)		
College and above	310(82.2)	67(17.8)		
Family economic level [*N*(%)]			8.962	0.011
<5,000 RMB/month	6,296(78.0)	1775(22.0)		
5,000–10,000 RMB/month	1,692(79.0)	449(21.0)		
>10,000 RMB/month	596(73.9)	210(26.1)		
Commuting to and from school [*N*(%)]			6.116	0.013
Positive approach	3,928(79.0)	1,045(21.0)		
Negative approach	4,656(77.0)	1,389(23.0)		
Sufficient sleep [*N*(%)]			21.257	<0.001
No (<8 h/day)	7,476(77.2)	2,204(22.8)		
Yes (≥8 h/day)	1,108(82.8)	230(17.2)		
Exercise duration [*N*(%)]			106.756	<0.001
<30 min/day	4,235(74.0)	1,486(26.0)		
30–60 min/day	3,444(82.6)	727(17.4)		
>60 min/day	905(80.4)	221(19.6)		
SSB consumption [*N*(%)]			90.086	<0.001
<1 times/week	3,051(80.0)	762(20.0)		
2–4 times/week	4,583(78.9)	1,228(21.1)		
>4 times/week	950(68.1)	444(31.9)		
Relative grip strength quartiles [*N*(%)]			34.674	<0.001
Q1	2032(73.9)	716(26.1)		
Q2	2,165(78.8)	583(21.2)		
Q3	2,222(79.0)	592(21.0)		
Q4	2,165(79.9)	543(20.1)		

N, numbers; SSB, sugar-sweetened beverage; BMI, body mass index.

Table 3 shows a one-way comparison of SSB consumption and relative grip strength with psychological symptoms among adolescents in rural areas of western China. Overall, the results showed that the differences in the prevalence of adolescents with different categories of SSB consumption in terms of the dimensions of emotional problems, behavioral problems, and social adjustment difficulties were statistically significant when compared with each other (*χ*^2^-values of 77.064, 68.132, 91.295, *p* < 0.001). The SSB consumption >4 times/week group had the highest prevalence of psychological symptoms and dimensions. The differences in the dimensions of emotional problems, behavioral problems, and social adjustment difficulties among adolescents with different relative grip strength quartiles were also statistically significant (*χ*^2^-values of 27.887, 27.431, and 26.431, *p* < 0.001). Relative grip strength quartiles were the highest prevalence of psychological symptoms and dimensions in adolescents in the Q1 group.

**Table 3:** A one-way comparison of SSB consumption and relative grip strength with psychological symptoms among adolescents in rural western China.

Group/Sex	*N*	Emotional problems			Behavioral problems			Social adjustment difficulties			Psychological symptoms		
		*N* (%)	*χ*^2^-value	*P-*value	*N* (%)	χ^2^-value	*P*-value	*N* (%)	χ^2^-value	*P*-value	*N* (%)	χ^2^-value	*P*-value
Boys													
SSB consumption [*N*(%)]			56.359	<0.001		31.801	<0.001		57.527	<0.001		42.081	<0.001
<1 times/week	1,530	424(27.7)			404(26.4)			304(19.9)			327(21.4)		
2–4 times/week	2,939	768(26.1)			767(26.1)			560(19.1)			612(20.8)		
>4 times/week	825	325(39.4)			295(35.8)			256(31.0)			258(31.3)		
Relative grip strength quartiles [*N*(%)]			22.005	<0.001		21.222	<0.001		16.408	0.001		23.592	<0.001
Q1	572	206(36.0)			202(35.3)			157(27.4)			171(29.9)		
Q2	881	269(30.5)			253(28.7)			191(21.7)			207(23.5)		
Q3	1,576	441(28.0)			425(27.0)			321(20.4)			355(22.5)		
Q4	2,265	601(26.5)			586(25.9)			451(19.9)			464(20.5)		
Girls													
SSB consumption [*N*(%)]			26.981	<0.001		39.784	<0.001		35.856	<0.001		50.076	<0.001
<1 times/week	2,283	607(26.6)			530(23.2)			383(16.8)			435(19.1)		
2–4 times/week	2,872	828(28.8)			779(27.1)			569(19.8)			616(21.4)		
>4 times/week	569	214(37.6)			205(36.0)			158(27.8)			186(32.7)		
Relative grip strength quartiles [*N*(%)]			13.313	<0.001		19.663	<0.001		25.637	<0.001		25.724	<0.001
Q1	2,176	682(31.3)			646(29.7)			493(22.7)			545(25.0)		
Q2	1867	529(28.3)			446(23.9)			333(17.8)			376(20.1)		
Q3	1,238	325(26.3)			313(25.3)			217(17.5)			237(19.1)		
Q4	443	113(25.5)			109(24.6)			67(15.1)			79(17.8)		
Total													
SSB consumption [*N*(%)]			77.064	<0.001		68.132	<0.001		91.295	<0.001		90.086	<0.001
<1 times/week	3,813	1,031(27.0)			934(24.5)			687(18.0)			762(20.0)		
2–4 times/week	5,811	1,596(27.5)			1,546(26.6)			1,129(19.4)			1,228(21.1)		
>4 times/week	1,394	539(38.7)			500(35.9)			414(29.7)			444(31.9)		
Relative grip strength quartiles [*N*(%)]			27.887	<0.001		27.431	<0.001		26.436	<0.001		34.674	<0.001
Q1	2,748	888(32.3)			848(30.9)			650(23.7)			716(26.1)		
Q2	2,748	798(29.0)			699(25.4)			524(19.1)			583(21.2)		
Q3	2,814	766(27.2)			738(26.2)			538(19.1)			592(21.0)		
Q4	2,708	714(26.4)			695(25.7)			518(19.1)			543(20.1)		

N, numbers; SSB, sugar-sweetened beverage.

Table 4 shows the logistic regression analysis of SSB consumption and relative grip strength with psychological symptoms among adolescents in rural areas of western China. The presence of psychological symptoms in adolescents was used as the dependent variable, and the SSB consumption and relative grip strength quartiles were analyzed as independent variables, respectively. Model 1 is crude, Model 2 adjusts age, father’s education level, mother’s education level, and family economic level based on Model 1, and Model 3 adjust commuting to and from school, sufficient sleep, exercise duration, and BMI based on Model 2. Overall, the results showed that the prevalence of psychological symptoms was significantly higher in adolescents in the SSB consumption >4 times/week group (OR = 1.95, 95% CI: 1.69–2.24), using the SSB consumption <1 time/week group as the reference group (*p* < 0.001). With relative grip strength quartiles as the Q4 group as the reference group, the prevalence of psychological symptoms in adolescents with relative grip strength quartiles in group Q1 (OR = 1.13, 95% CI: 1.69–2.24) was also significantly higher, but the results were not significant.

**Table 4:** Logistic regression analysis of SSB consumption and relative grip strength with psychological symptoms among adolescents in rural western China.

Sex/variable	Group	Psychological symptoms					
		Model 1		Model 2		Model 3	
		OR (95% CI)	*P-*value	OR (95% CI)	*P-*value	OR (95% CI)	*P-*value
Boys							
SSB consumption	<1 times/week	1.00		1.00		1.00	
	2–4 times/week	0.97(0.83 ~ 1.13)	0.669	0.97(0.83 ~ 1.13)	0.674	1.01(0.87 ~ 1.18)	0.921
	>4 times/week	1.67(1.38 ~ 2.03)	<0.001	1.68(1.38 ~ 2.03)	<0.001	1.71(1.41 ~ 2.08)	<0.001
Relative grip strength quartiles	Q4	1.00		1.00		1.00	
	Q3	1.13(0.97 ~ 1.32)	0.129	1.11(0.95 ~ 1.30)	0.208	1.06(0.90 ~ 1.24)	0.500
	Q2	1.19(0.99 ~ 1.44)	0.064	1.14(0.95 ~ 1.38)	0.167	1.06(0.87 ~ 1.29)	0.580
	Q1	1.66(1.35 ~ 2.03)	<0.001	1.57(1.27 ~ 1.94)	<0.001	1.34(1.06 ~ 1.69)	0.013
Girls							
SSB consumption	<1 times/week	1.00		1.00		1.00	
	2–4 times/week	1.16(1.01 ~ 1.33)	0.034	1.15(1.00 ~ 1.32)	0.050	1.19(1.04 ~ 1.37)	0.013
	>4 times/week	2.06(1.68 ~ 2.53)	<0.001	2.07(1.69 ~ 2.54)	<0.001	2.17(1.76 ~ 2.67)	<0.001
Relative grip strength quartiles	Q4	1.00		1.00		1.00	
	Q3	1.09(0.82 ~ 1.45)	0.545	1.11(0.83 ~ 1.46)	0.489	1.06(0.8 ~ 1.41)	0.674
	Q2	1.16(0.89 ~ 1.52)	0.273	1.17(0.89 ~ 1.53)	0.258	1.07(0.81 ~ 1.40)	0.643
	Q1	1.54(1.19 ~ 2.00)	0.001	1.54(1.19 ~ 2.00)	0.001	1.26(0.96 ~ 1.66)	0.091
Total							
SSB consumption	<1 times/week	1.00		1.00		1.00	
	2–4 times/week	1.07(0.97 ~ 1.19)	0.174	1.07(0.96 ~ 1.18)	0.218	1.11(1.01 ~ 1.24)	0.039
	>4 times/week	1.87(1.63 ~ 2.15)	<0.001	1.87(1.63 ~ 2.15)	<0.001	1.95(1.69 ~ 2.24)	<0.001
Relative grip strength quartiles	Q4	1.00		1.00		1.00	
	Q3	1.06(0.93 ~ 1.21)	0.365	1.05(0.92 ~ 1.20)	0.433	0.99(0.87 ~ 1.13)	0.918
	Q2	1.07(0.94 ~ 1.22)	0.288	1.06(0.93 ~ 1.21)	0.417	0.95(0.83 ~ 1.09)	0.475
	Q1	1.41(1.24 ~ 1.60)	<0.001	1.38(1.21 ~ 1.57)	<0.001	1.13(0.99 ~ 1.29)	0.080

SSB, sugar-sweetened beverage; OR, Odds Ratio; 95% CI, 95% Confidence Interval. Model 1 was crude, Model 2 was adjusted for age, father’s education level, mother’s education level, and family economic level based on Model 1, and Model 3 was adjusted for commuting to and from school, sufficient sleep, exercise duration, and BMI based on Model 2.

**Figure 2:**
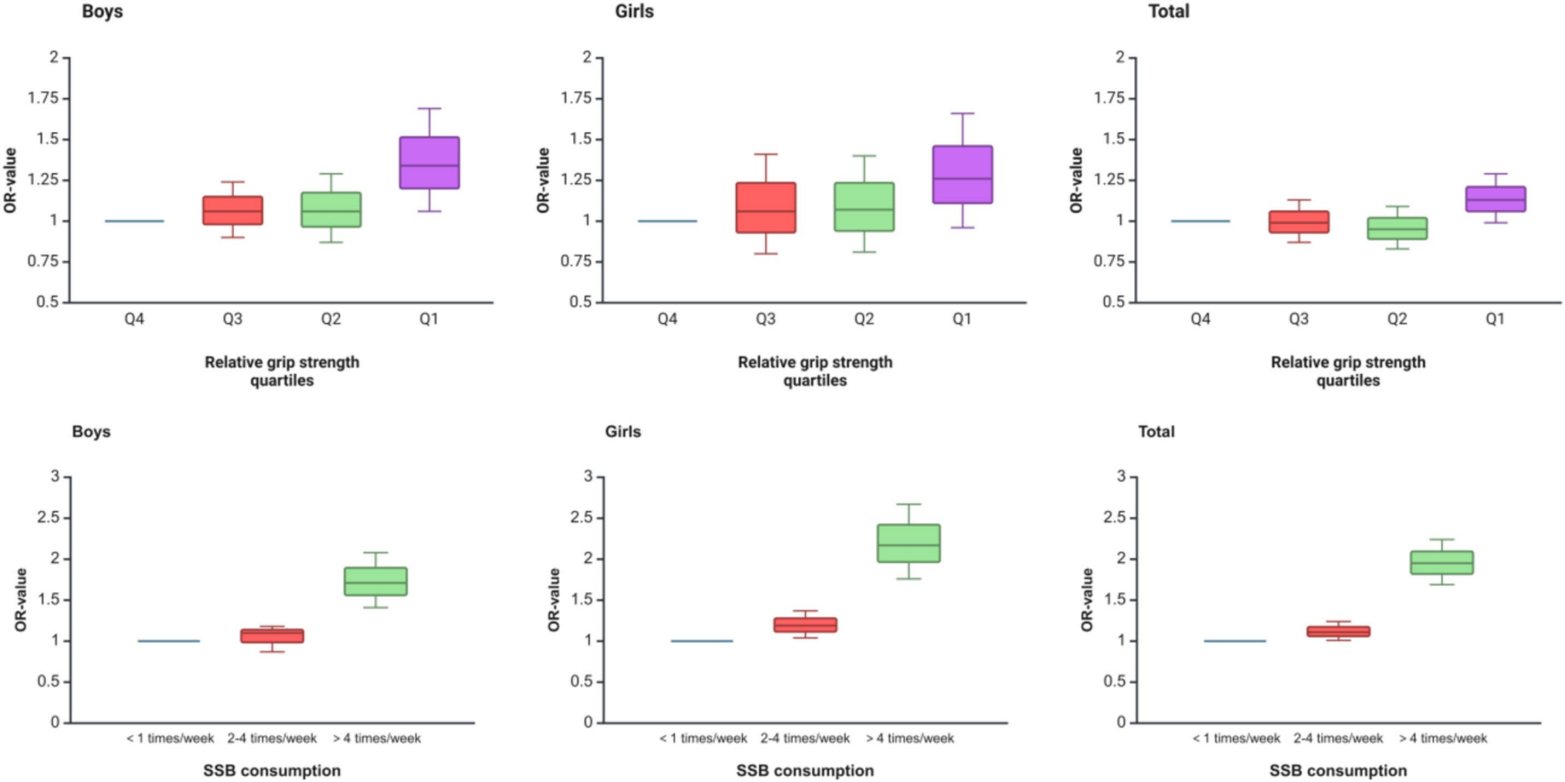
Trends in ORs of logistic regression analysis of SSB consumption and relative grip strength with psychological symptoms among adolescents in rural areas of western China.

Figure 2 shows the trend of OR values of logistic regression analysis of SSB consumption and relative grip strength with psychological symptoms in adolescents in rural areas of western China. As can be seen from the figure, with the increase in SSB consumption and the decrease in relative grip strength, adolescents had a higher risk of developing psychological symptoms, and the OR value was larger.

Table 5 shows the ordered logistic regression analysis of SSB consumption and relative grip strength with psychological symptoms among adolescents in rural areas of western China. Ordered logistic regression analyses with generalized linear models were conducted using the presence of psychological symptoms among adolescents in rural areas of western China as the dependent variable, and the joint quartiles of SSB consumption and relative grip strength as the independent variables. Ordered logistic regression Model adjusts age, father’s education level, mother’s education level, family’s economic level, commuting to and from school, sufficient sleep, exercise duration, and BMI. Overall, the results of the analysis showed that, using the SSB consumption <1 times/week group and relative grip strength quartiles as the Q4 group as the reference group. Adolescents in the SSB consumption >4 times/week group and relative grip strength quartiles as Q1 group (OR = 2.77, 95% CI: 2.09–3.67) had the highest risk of psychological symptoms (*p* < 0.001).

**Table 5:** Ordered logistic regression analysis of SSB consumption and relative grip strength with psychological symptoms among adolescents in rural western China.

Sex	Classification of interaction		Psychological symptoms	
	SSB consumption	Relative grip strength quartiles	*OR* (95% *CI*)	*P*-value
Boys	<1 times/week	Q4	1.00	
		Q3	1.25(0.94 ~ 1.67)	0.122
		Q2	0.99(0.69 ~ 1.42)	0.949
		Q1	1.17(0.76 ~ 1.81)	0.475
	2–4 times/week	Q4	0.92(0.73 ~ 1.16)	0.489
		Q3	1.00(0.78 ~ 1.28)	0.994
		Q2	1.13(0.85 ~ 1.51)	0.384
		Q1	1.64(1.21 ~ 2.22)	0.001
	>4 times/week	Q4	1.58(1.16 ~ 2.15)	0.003
		Q3	1.63(1.17 ~ 2.27)	0.004
		Q2	2.02(1.38 ~ 2.95)	<0.001
		Q1	2.85(1.86 ~ 4.36)	<0.001
Girls	<1 times/week	Q4	1.00	
		Q3	0.95(0.61 ~ 1.48)	0.815
		Q2	1.04(0.68 ~ 1.59)	0.846
		Q1	0.97(0.63 ~ 1.49)	0.889
	2–4 times/week	Q4	0.97(0.63 ~ 1.49)	0.889
		Q3	0.97(0.63 ~ 1.49)	0.889
		Q2	1.09(0.72 ~ 1.65)	0.675
		Q1	1.40(0.63 ~ 3.13)	0.411
	>4 times/week	Q4	1.40(0.63 ~ 3.13)	0.411
		Q3	2.03(1.20 ~ 3.42)	0.008
		Q2	1.75(1.07 ~ 2.88)	0.026
		Q1	2.95(1.85 ~ 4.73)	<0.001
Total	<1 times/week	Q4	1.00	
		Q3	1.06(0.84 ~ 1.33)	0.642
		Q2	0.97(0.77 ~ 1.22)	0.764
		Q1	1.06(0.85 ~ 1.33)	0.607
	2–4 times/week	Q4	0.92(0.74 ~ 1.14)	0.446
		Q3	0.97(0.78 ~ 1.19)	0.748
		Q2	1.05(0.85 ~ 1.30)	0.651
		Q1	1.50(1.22 ~ 1.84)	<0.001
	>4 times/week	Q4	1.58(1.19 ~ 2.10)	0.002
		Q3	1.73(1.32 ~ 2.28)	<0.001
		Q2	1.80(1.35 ~ 2.39)	<0.001
		Q1	2.77(2.09 ~ 3.67)	<0.001

SSB, sugar-sweetened beverage; OR, Odds Ratio; 95% CI, 95% Confidence Interval. Ordered logistic regression Model adjusts age, father’s education level, mother’s education level, family’s economic level, commuting to and from school, sufficient sleep, exercise duration, and BMI.

**Figure 3:**
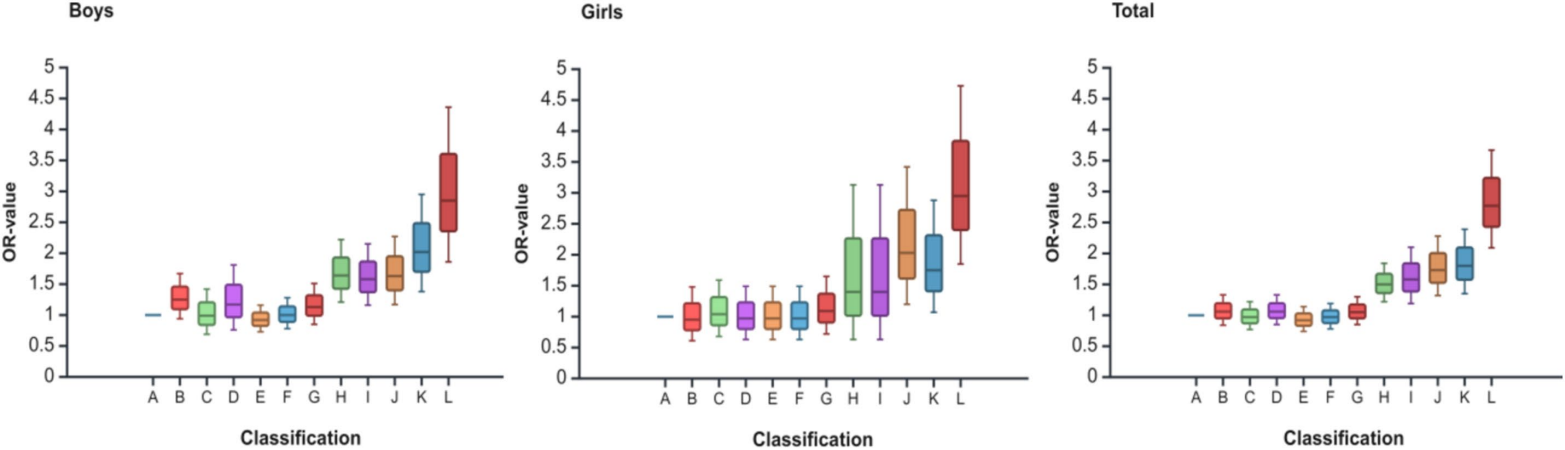
Trends in ORs of ordered logistic regression analyses of SSB consumption and relative grip strength with psychological symptoms among adolescents in rural areas of western China. “times/week” is SSB consumption; “Q” is relative grip strength quartiles. A is <1 times/week and Q4; B is <1 times/week and Q3; C is <1 times/week and Q2; D is <1 times/week and Q1; E is 2–4 times/week and Q4; F is 2–4 times/week and Q3; G is 2–4 times/week and Q2; H is 2–4 times/week and Q1; I is >4 times/week and Q4; J is >4 times/week and Q3; K is >4 times/week and Q2; L is >4 times/week and Q1.

Figure 3 shows the trend of OR values of ordered logistic regression analysis of SSB consumption and relative grip strength with psychological symptoms among adolescents in rural areas of western China. As can be seen from the figure, as SSB consumption increases and relative grip strength decreases, adolescents have a higher risk of developing psychological symptoms, i.e., the OR value is higher, up to 2.77.

## 4 Discussion

The prevalence of psychosomatic symptoms among adolescents is rising steadily, negatively affecting the health of adolescents and future adults, and placing a serious disease burden on the country (45). To the best of our knowledge, this study is the first to assess the prevalence of psychological symptoms and their association with SSB consumption and relative grip strength among adolescents in rural areas of western China. The results of this study showed that the prevalence of psychological symptoms among adolescents in rural areas of western China was 22.1%. This result is higher than that of the national survey on the prevalence of psychological symptoms among adolescents in China (16.3%) (46). It is also higher than the results of the survey on adolescents’ psychological symptoms in Eastern China (21.39%) (47). It can be seen that the prevalence of psychological symptoms among adolescents in the rural areas of western China is high, which should be paid attention to and analyzed to reduce the prevalence of psychological symptoms among adolescents in the rural areas of western China. Overall, the reasons for the higher prevalence of adolescent psychological symptoms in rural areas of western China are manifold. First, compared with the eastern part of China, the western part of the country is relatively backward in terms of economic development, and the investment in education and medical care is relatively low, which leads to limited attention and financial investment in mental health education by families and schools, and is an important reason for the higher prevalence of adolescent psychological symptoms in the western rural areas. Research shows that the incidence of adolescent mental health problems in economically backward areas is higher than that in economically more developed areas (48). Second, the relatively low educational level of parents in rural areas of western China, who do not pay enough attention to their children’s mental health, coupled with the fact that more parents go out to work and have less time to accompany and educate their children, is another important reason for the high prevalence of psychological symptoms among adolescents in rural areas of western China. Past studies have shown that the higher the level of parental education, the lower the risk of adolescents developing psychological problems, showing a negative correlation (49).

The present study also showed that the proportion of adolescents with SSB consumption >4 times/week in rural areas of western China was 12.7%, suggesting that a certain proportion of adolescents have high SSB consumption, which should be a cause for concern. The specific reason is related to the prevalence of short videos and various kinds of publicity advertisements brought about by the development of the current information network and the development of cell phone smart terminals. The lack of health knowledge among adolescents or guardians in remote rural areas is an important reason for the higher consumption of SSB. A survey of SSB consumption among Spanish adolescents showed that the proportion of those with SSB consumption higher than 3 times/week was 9.6%, which was lower than the results of this study (50). Surveys have also shown that SSB consumption among adolescents in rural areas of China is increasing, suggesting that health education and guidance should be given to adolescents in rural areas to improve their health literacy, reduce SSB consumption, and promote physical and mental health (51). The results of this study also showed that adolescents with higher SSB consumption in rural areas of western China were at higher risk of developing psychological symptoms. A study has shown that higher SSB consumption tends to lead to the development of overweight or obesity, and the detection rate of psychological symptoms is relatively high among obese individuals (52). Studies have also shown that high SSB consumption can lead to intestinal flora disorders, inflammation of the nervous system, and hormonal abnormalities, which can lead to various psychological disorders (53). The results of this study also showed that lower relative grip strength was associated with a higher risk of developing psychological symptoms among adolescents in rural areas of western China. Previous studies have found that adolescents with lower muscle strength are associated with lower physical activity and shorter duration of exercise, which are strongly associated with the development of psychological symptoms (54). It has also been shown that lower muscle strength in adolescents leads to elevated levels of inflammation in the body and that elevated levels of inflammation are associated with the development of psychological symptoms (55).

The joint effect analysis of SSB consumption and relative grip strength in this study showed that adolescents with higher SSB consumption and lower relative grip strength had a higher risk of developing psychological symptoms in rural areas of western China, indicating that there was a joint effect of SSB consumption and relative grip strength on psychological symptoms. Previous studies have also found that increased SSB consumption is associated with decreased muscle strength and that increased SSB consumption may hinder muscle neuron regeneration, which may hurt muscle strength (56). It has also been shown that increased SSB consumption in adolescents leads to the development of obesity and that obese individuals have relatively low muscle strength (21). In addition to this, it has also been found that increased SSB consumption can lead to inflammation in the body, as well as a decrease in the body’s muscle strength, which can have a detrimental effect on health (57). The results of this study also suggest that in the future, we should take necessary measures to reduce SSB consumption and improve muscle strength in adolescents in rural areas of western China to better promote adolescent mental health development.

The present study has some strengths. First, to the best of our knowledge, this study is the first to investigate the association between SSB consumption and relative grip strength with psychological symptoms in adolescents in rural areas of western China. This study may provide necessary help for the reduction and intervention of adolescents’ psychological symptoms in rural areas of western China. Second, this study assessed SSB consumption, relative grip strength, and psychological symptoms among adolescents in Xinjiang and Tibet in the rural areas of western China, which has typical regional characteristics. This study enriches the basic information in the field of adolescent mental health in rural areas of western China and provides theoretical support for the development of adolescent physical and mental health in rural areas of western China. However, there are some limitations to this study. First, the present study was a cross-sectional investigation and was only able to analyze the associations between SSB consumption and relative grip strength with psychological symptoms, but not the causal associations that existed between them. Prospective cohort studies should be conducted in the future to compensate for this deficiency. Second, although this study investigated Xinjiang and Tibet in western China, Gansu, and Ningxia, which are also in the western part of China, were not investigated and evaluated due to the vastness of China. In the future, more surveyed regions should be included to improve the typicality and representativeness of the study.

## 5 Conclusion

The prevalence of psychological symptoms among adolescents in rural areas of western China was high compared with that of the whole country. Adolescents’ SSB consumption and relative grip strength were associated with psychological symptoms. The prevalence of psychological symptoms was higher in adolescents with higher SSB consumption and lower relative grip strength. Prospective cohort studies are needed in the future to explore the causal relationships among SSB consumption, muscle strength, and psychological symptoms.

## Data Availability

The raw data supporting the conclusions of this article will be made available by the authors, without undue reservation.
